# The concrete evidence of flexistyly in *Plagiostachys*: pollination biology of a wild ginger on Hainan Island, China

**DOI:** 10.1002/ece3.1807

**Published:** 2015-10-30

**Authors:** Xiao‐Cheng Jia, Jia Li, Guo‐Hui Lu, Ying‐Qiang Wang

**Affiliations:** ^1^ Hainan Key Laboratory of Tropical Oil Crops Biology/Coconut Research Institute Chinese Academy of Tropical Agricultural Sciences Wenchang Hainan 571339 China; ^2^ College of Life Science South China Normal University Guangzhou 510631 China; ^3^ Guangdong Provincial Key Lab of Biotechnology for Plant Development South China Normal University Guangzhou 510631 China

**Keywords:** Flexistyly, *Plagiostachys austrosinensis* T. L. Wu & S. J. Chen, pollination biology, Zingiberaceae

## Abstract

Flexistyly in *Plagiostachys* was first reported by Takano et al., while they provided no detailed study on pollination biology and breeding system. In this study, we tested this suspicion in one species of *Plagiostachys* (*Plagiostachys austrosinensis*). Field observations suggested that flexistyly was present in this species, and stigmatic behavior was similar to that reported for *Alpinia* and *Amomum* species. Two phenotypes (anaflexistylous and cataflexistylous) occurred in a ratio of 1:1 in natural populations. Anthesis began around 1530–1600 h and lasted for about 24 h. Pollen viability and stigma receptivity remained high throughout the flowering process. Mean nectar volume (4.15–11.30 *μ*L) and mean sugar concentration (>32%) also remained at a high level during the flowering process. No fruit set occurred in unpollinated bagged plants. Two pollinators (*Bombus pyrosoma* and Vespidae spp.) and one pollen robber (Mutillidae spp.) were found as flower visitors. Fruit set following self‐pollination and cross‐pollination did not differ significantly in the cataflexistylous morph. Partial self‐incompatibility was apparent in the anaflexistylous morph. These results provide the concrete evidence of flexistyly in *Plagiostachys* and a more thorough understanding of its evolutionary origin in gingers.

## Introduction

Understanding how sexual diversity evolves and how it is maintained is of central interest in ecology and evolutionary biology and receives great public awareness. Most adaptive interpretations of mating system have focused on mechanisms that promote outcrossing and reduce the likelihood of inbreeding depression (Eckert and Barrett 1994; Barrett 2002). Floral dimorphisms, such as heterostyly, monoecy, dioecy, and heterodichogamy, have been considered as mechanisms to enhance outcrossing (Barrett 2003).

Flexistyly is a unique floral dimorphism achieved by changing the position of the style over time (Cui et al. 1995; Li et al. 2001a; Zhang et al. 2003; Takano et al. 2005; Ren et al. 2007; Takano et al. 2009). A flexistylous population comprises two types of floral morphs, termed cataflexistylous (protandrous) and anaflexistylous (protogynous) morphs, and both types reciprocally change from one sexual phase to the other in the middle of the 1‐day flowering period (Li et al. 2001b). When cataflexistyled flowers are open, the stigma is held above the open anther from which pollen is being released. At the same time of day, the receptive stigma of anaflexistyled flowers is curved downwards, below the indehiscent anther from which pollen has not yet been shed. Flowers of both types retain these respective stigma positions until about midday, when the stigma of the anaflexistyle form elongates and becomes erect above the anther. The anther then dehisces and pollen is released. In the cataflexistyled flower, the stigma begins to move downwards and enter the receptive position. This floral dimorphism was first discovered in *Amomum tsao‐ko* Crevost & Lemarie (Cui et al. 1995) and then in species of *Alpinia*,* Etlingera,* and *Plagiostachys* (Li et al. 2001a; Zhang et al. 2003; Kress et al. 2005; Ren et al. 2007; Takano et al. 2009).

The family Zingiberaceae (ginger family), with ca. 1300 species (Larsen et al. 1998), is characterized by diverse floral forms and specialized long‐tubed flowers. It is one of the most diverse plant groups in the Asian tropics (Zhang et al. 2014, 2015; Bhadra and Bandyopadhyay 2015). Furthermore, it exhibits various sexual syndromes (Sakai et al. 2013; Yang et al. 2014), including andromonoecy in *Amomum dimorphum* (Sakai and Nagamasu 1998), heterodichogamy in *Alpinia* species (Barrett 2002), and autogamy in *Caulokaempferia coenobialis* (Wang et al. 2004a). The main pollinators of this family are long‐tongued animals, including Aves, Hymenoptera, and Lepidoptera (Ippolito and Armstrong 1993; Kato et al. 1993; Sakai et al. 1999). Morphological characters of zingiberaceous flowers are thought to have coevolved with their pollinators (Kato et al. 1993).

**Figure 1:**
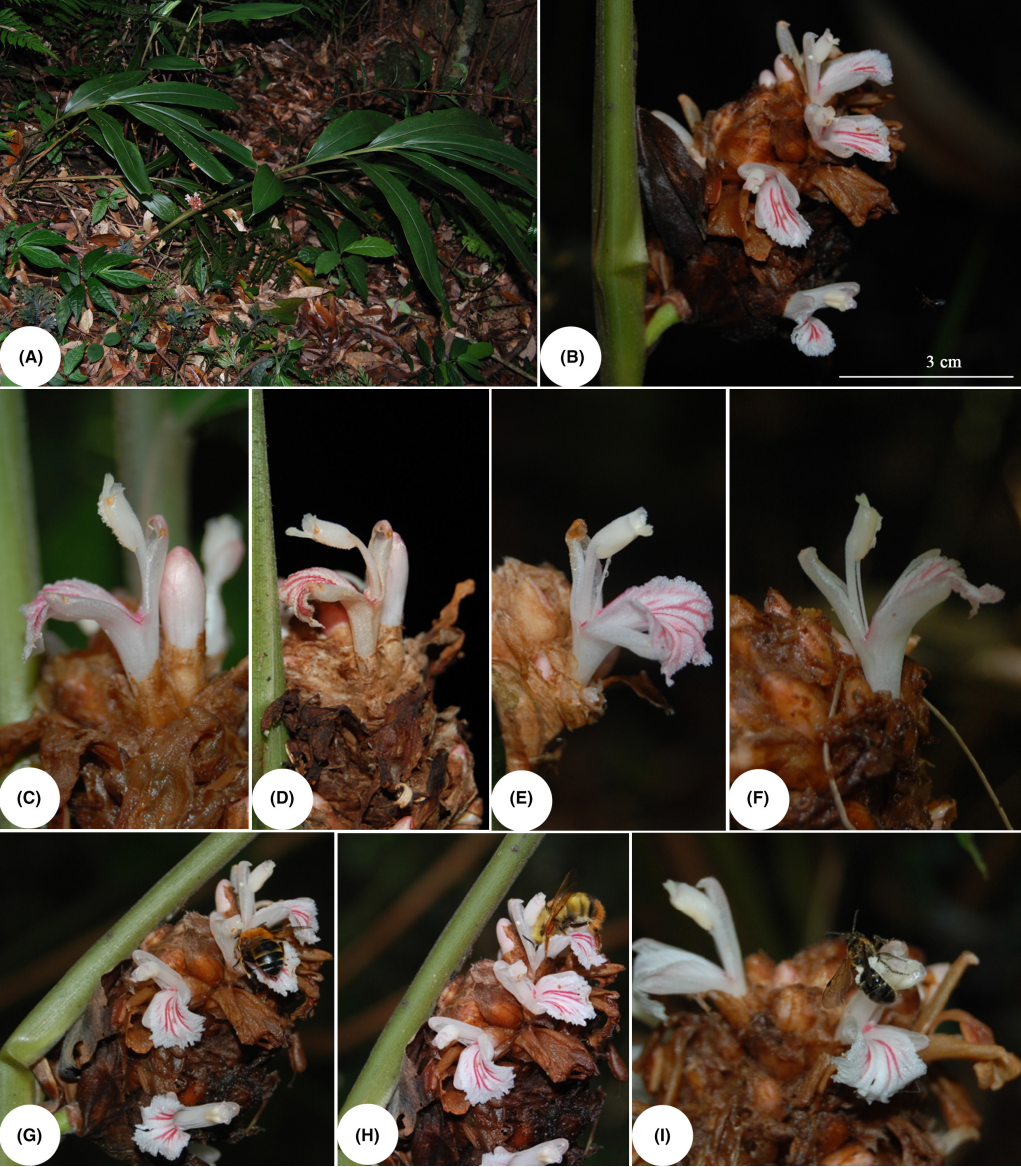
*Plagiostachys austrosinensis* and flower visitors (A) Individual plant; (B) Inflorescence; (C) Cataflexistylous flower in the 1st stage; (D) Cataflexistylous flower in the 2nd stage; (E) Anaflexistylous flower in the 1st stage; (F) Anaflexistylous flower in the 2nd stage; (G) Pollinator (Vespidae spp.); (H) Pollinator (*Bombus pyrosoma* Morawitz); (I) Pollen robber (Mutillidae spp.).

*Plagiostachys* Ridl. is a relatively small but complex genus in Zingiberaceae. It is distributed mainly in the Malesian region with its center of diversity in Borneo, where ten species of *Plagiostachys* are currently known (Julius et al. 2008). The genus is distinguished from other genera in Zingiberaceae by its tightly congested, apparently lateral inflorescences, which are actually terminal on short stems of the leafy shoots and usually break through the leaf sheaths above ground level or sometimes in the middle (Smith 1990). The flower is subtended by a usually tubular bracteole and the labellum is small and rather fleshy, with diverged petaloid venation in some species (Smith 1985). *Plagiostachys* plants are frequently visited by bees and spiderhunters (Sakai et al. 1999). A single plant usually flowers more than once a year and flowering is not synchronized among individuals (Sakai 2000). *Plagiostachys austrosinensis* T. L. Wu & S. J. Chen, the only Chinese species in the genus, is a perennial herb. It is endemic to Guangdong, Guangxi, and Hainan provinces in southern China (Wu and Larsen 2000). This plant is immediately recognizable by its conical inflorescence, which breaks through the leaf sheath about 14–16 cm above the ground (Fig. 1A and B).

Kress et al. (2005) state that flexistyly has evolved in the common ancestor of the tribe Alpinieae or has independently occurred at least three to five times in the tribe. Reproductive biology, including the pollination of flexistylous gingers, has been studied in at least 24 species, including *Amomum tsao‐ko* (Cui et al. 1995), *Amomum maximum* (Ren et al. 2007), *Alpinia kwangsiensis* (Li et al. 2002), *Alpinia blepharocalyx* (Zhang et al. 2003), *Alpinia nieuwenhuizii* (Takano et al. 2005), *Etlingera yunnanensis* (Kress et al. 2005), *Plagiostachys strobilifera* (Takano et al. 2009), and possibly *Paramomum* species (Cui et al. 1996). To investigate the evolutionary origin and significance of flexistyly, it is essential to clarify the prevalence and distribution of flexistyly within the family. Furthermore, the biology of different flexistylous species in different habitats should also be studied.

In this study, we report flexistyly in *Plagiostachys austrosinensis* T. L. Wu & S. J. Chen at Li‐mu‐shan Mountain, a National Forestry Park in Hainan province. Although the floral behavior was similar to that of previously reported flexistylous species, strikingly different behaviors of two pollinators and one pollen robber were observed in this study. In addition, differences in fruit set between self‐ and cross‐fertilized treatments indicate partial self‐incompatibility in the anaflexistylous morph. To further increase our understanding of the evolution of flexistyly itself, we addressed the following questions: (1) Is the stigma behavior of the *Plagiostachys austrosinensis* flowers the same as in other reported flexistylous gingers during anthesis? (2) What are the patterns of nectar secretion, sugar concentration and production, and pollinator visiting frequency? (3) What are the characteristics of the breeding systems?

## Materials and Methods

### Species and study site

*Plagiostachys austrosinensis* T. L. Wu & S. J. Chen, the only Chinese species in the genus *Plagiostachys*, is a perennial herb about 50–100 cm in height belonging to the family Zingiberaceae. One population of *Plagiostachys austrosinensis* was studied at the summit of Li‐mu‐shan Mountain (LMS) (E109°45′59.7′′, N19°10′52.3′′, 1386 m elev.), a National Forestry Park in Hainan province. The population comprised about 5000 plants. Plants were visited repeatedly between May and June in 2013 and 2014, confirming that the species flowers between May and June (Table 1). Field observations and experimental samples were collected from 1 May to 15 June 2013 and from 2 May to 16 June 2014. Specimens and floral visitors were deposited at the Institute of Medicinal Plant Development, Chinese Academy of Medical Sciences and Peking Union Medical College.

**Table 1:** Flowering phenology of *Plagiostachys austrosinensis* in the Li‐mu‐shan Biosphere Reserve

Latitude, longitude, and altitude	Onset of flowering	Peak of flowering	Flowering termination
E109°45′59.7′′ N19°10′52.3′′ ALT: 1386 m	03‐May‐2013 01‐May‐2014	12‐May‐2013 17‐May‐2014	13‐June‐2013 15‐June‐2014

### Flower phenology

Flower phenology observations were carried out on 25 and 28 April and 1, 3, 5, 12, 15, 16, and 17 May and 10, 13, and 16 June 2013 and 25 and 29 April and 1, 3, 5, 12, 15, and 17 May and 10, 15, and 18 June 2014, for a total of 23 days. In each year of 2013 and 2014, 40 buds (20 anaflexistylous plants + 20 cataflexistylous plants) from different individuals were selected and tagged. The flower phenology of these buds was then observed and recorded.

### Pollen and stigma morphology and P/O ratio

Flower morphology was studied under a stereomicroscope (Zeiss 2000‐C: Jena, Germany). Surface details of pollen and stigmas were later observed under a scanning electron microscope (SEM) using conventional methods. The pollen/ovule ratio (P/O) was evaluated in ten pre‐anthesis flower buds following Wang et al. (2004b).

### Measurement of nectar volume and sugar concentration

In 2013 and 2014, we randomly selected 10 (5 ana‐flowers + 5 cata‐flowers) freshly opened flowers and extracted their nectar using 5‐ or 10‐*μ*L capillary tubes every 2 h between 0800 and 1830 h. Nectar sugar concentration was measured with a handheld temperature‐compensated refractometer (ATAGO N‐50E: Tokyo, Japan).

### Pollen viability and stigma receptivity

Stigma receptivity and pollen viability were evaluated in five flowers collected every 2 h (from 1600 to 1830 on the first day and from 0800 to 1600 on the second day, see Figure 3) during the flowering stage. MTT (3‐(4,5‐Dimethylthiazol‐2‐yl)‐2,5‐diphenyltetrazolium bromide) was used to stain the stigma and pollen grains. Dark or brown spots indicate the presence of dehydrogenase, reflecting receptivity and viability (Dafni 1992).

### Pollinator observation

Pollinator observations were carried out on 15, 16, and 17 May 2013 and 13, 14, and 15 May 2014 between 0700 and 1800 h, for a total of 52 h. We recorded the number of flowers visited and whether the flower visit was legitimate (i.e., whether anthers or stigmatic lobes were touched). All flower visitors and their visiting times and handling times were recorded, and insects were then captured for identification.

### Breeding system experiments

In 2013 and 2014, we performed the following pollination treatments at LMS: (1) open pollination: buds were marked before anthesis and then left exposed; (2) agamospermy A: stamens were removed from buds just before anthesis, and buds were then enclosed in bags; (3) agamospermy B: stigmas were removed from buds just before anthesis, and buds were then enclosed in bags; (4) automatic selfing (bagged flowers): buds about to open were enclosed in bags; (5) experimental selfing: freshly opened flowers were hand‐pollinated with self‐pollen and then enclosed in bags; (6) experimental outcrossing: pollen from plants at least 100 m away was placed directly onto the stigmas of freshly opened flowers. Cata‐crossing (cataflexistylous ♀ **×** anaflexistylous ♂) and ana‐crossing (anaflexistylous ♀ **×** cataflexistylous ♂) were conducted reciprocally. We bagged the inflorescences of experimental plants prior to anthesis with a coarse mesh bag that prevented transfer of pollen. Subsequently, all remaining unpollinated flowers on each inflorescence were removed.

### Data analysis

Statistical analyses were performed using SPSS (version 19.0): IBM, Armonk, USA. GLM and MANOVA were used to compare fruit sets between cataflexistylous and anaflexistylous flowers. A value of *P* < 0.05 was accepted as significant difference. Data on the pollen/ovule ratio (P/O) were also compared between the two morphs using a *t*‐test of means. Results are given as mean ± SD.

## Results

### Flower phenology

In the studied population of *P. austrosinensis*, two morphs (cataflexistyly and anaflexistyly) are present in a ratio of 1:1. Inflorescences of the two floral morphs bloom at almost the same time and last about 1 month, from May to June (Table 1). Each inflorescence has a total of 15 to 60 (33.4 ± 5.4, *n* = 50) flowers and 1 to 5 (3.21 ± 1.01, *n* = 25) flowers blooming each day during anthesis. Anthesis begins around 1530–1600 h. In cataflexistylous flowers, the stigma is held erect above the dehisced anther when anthesis begins (Fig. 1C) and becomes curved under the anther at 1230 to 1300 on the second day (Fig. 1D). In anaflexistylous flowers, the stigma is first curved under the undehisced anther (Fig. 1E) and moves into a reflexed superior position above the anther as it begins to shed pollen at 1300 h on the second day (Fig. 1F). By 1530–1600 h on the second day, flowers begin to collapse and anthesis ends. The duration of anthesis is about 24 h. Bracts, calyces, dorsal lobes, ventral lobes, and lateral staminodes show no differences between cataflexistylous and anaflexistylous flowers during flowering (Fig. 1C–F). Both flower morphs have a high P/O ratio, 526.67 ± 101.36 (*n* = 10) for anaflexistylous flowers and 533.46 ± 92.37 (*n* = 10) for cataflexistylous flowers; these ratios are not significantly different (*t*‐test, *P* = 0.45).

### Pollen morphology and viability and stigma receptivity

**Figure 2:**
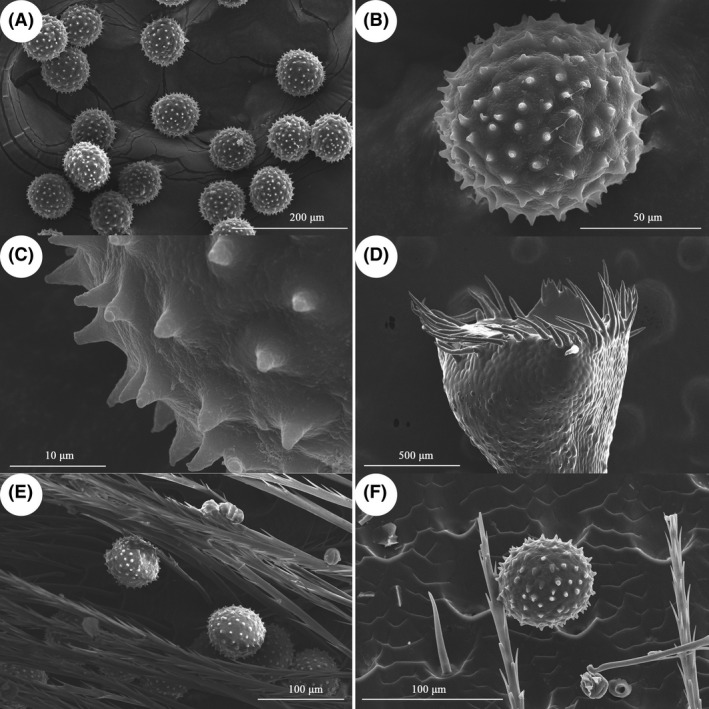
Morphological characters of pollen and stigma of *Plagiostachys austrosinensis* (A) Pollens (SEM); (B) Individual pollen (SEM); (C) Pollen surface sculpture (SEM); (D) Stigma (SEM); (E) Pollen grains on the back of *Bombus pyrosoma* Morawitz (SEM); (F) Pollen grains on the back of Vespidae spp. (SEM).

**Figure 3:**
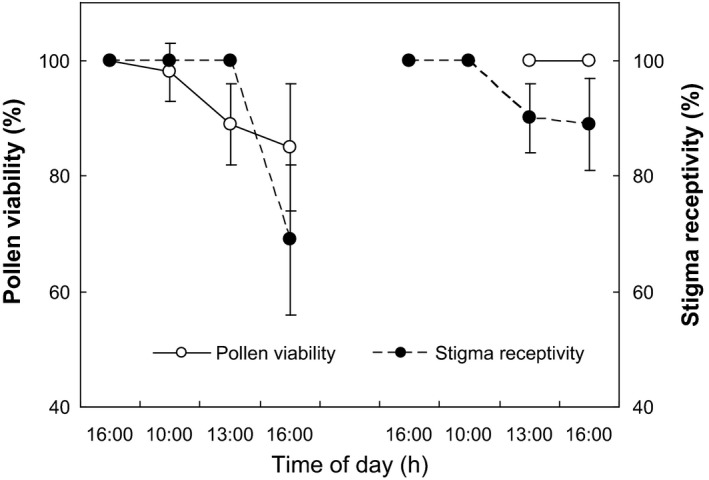
Pollen viability and stigma receptivity of *Plagiostachys austrosinensis* during flowering process (Left: cataflexistyly, Right: anaflexistyly; mean ± SD, *n* = 10).

Pollen grains are spheroidal with spur‐shaped sculpting (Fig. 2A–C). Stigmas are capitate with long hairs along the surface (Fig. 2D). Pollen viability varies from 85% to 100% (Fig. 3). Stigma receptivity remains at 100% during anthesis and begins to decrease on the second day (Fig. 3). Pollen viability remains high (>90%) up to 1200 h but declines slowly on the second day. By 1600 h on the second day, when cataflexistylous flowers have collapsed, pollen viability declines to 85% (Fig. 3).

### Nectar volume and sugar concentration

**Figure 4:**
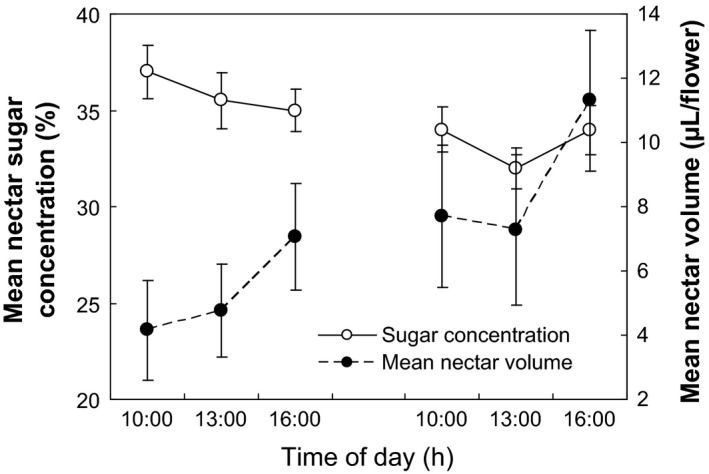
Mean nectar volume and sugar concentration of *Plagiostachys austrosinensis* (Left: cataflexistyly, Right: anaflexistyly; mean ± SD,* n* = 10).

Mean nectar volume remains high (4.15–11.30 *μ*L) during the flowering process (Fig. 4). The peak nectar volume is reached at 1630 h on the second day, declining quickly when flowers collapse (Fig. 4). Mean sugar concentration also remains high (>32%) during the flowering process. The peak sugar concentration is reached at 1600 h on the first day, declining slowly on the second day (Fig. 4).

### Flower visitors

**Figure 5:**
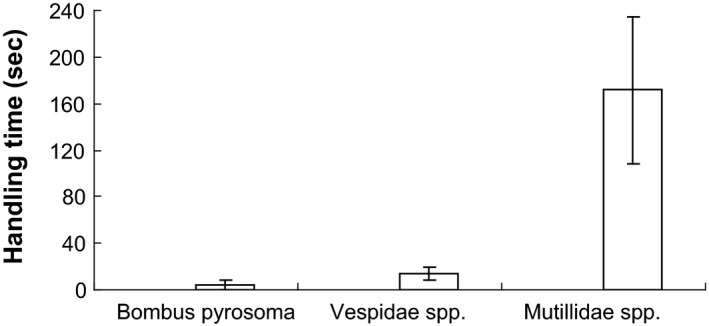
Comparison of handling time of flower visitors of *Plagiostachys austrosinensis* (mean ± SD,* n* = 10).

**Figure 6:**
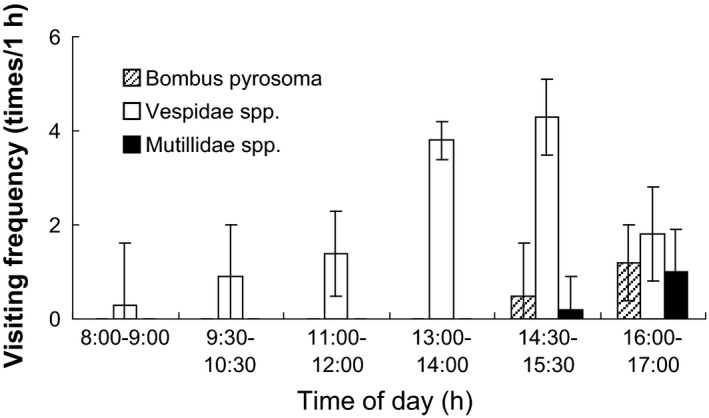
The visiting frequency of visitors of *Plagiostachys austrosinensis* (mean ± SD,* n* = 10).

During the 52 h of observation, two pollinators (*Bombus pyrosoma* and Vespidae spp.) and one pollen robber (Mutillidae spp.) were found as flower visitors (Fig. 1G–I). *Bombus pyrosoma* and Vespidae spp. occasionally crawled onto the flower, sucking nectar by inserting their long mouthparts into the corolla tube. Pollen grains were found on the backs of these two types of visitors (Fig. 2E and F). They were all considered effective pollinators. Ants were also found to forage for pollen masses and left quickly. Mutillidae spp. was considered a pollen robber, as it did not exhibit pollination behaviors such as nectar consumption, stigma touching, and grooming. The pollen robber stayed on flowers for a significantly longer time than the pollinators did (Fig. 5). Vespidae spp. was considered a frequent visitor; its peak visiting frequency occurred from 1430 to 1530 h on the second day (Fig. 6).

### Breeding system

Results of the breeding system experiments are shown in Table 2. In cataflexistylous flowers, fruit set following self‐pollination and cross‐pollination did not differ significantly (Table 2). In anaflexistylous flowers, there is significantly more fruit set following cross‐pollination than that following open pollination and self‐pollination (Table 2). The species is incapable of asexual seed set, as shown by the absence of seed set in flowers that had their stamens or stigmas cutoff before they could self‐pollinate (Table 2).

**Table 2:** Fruit set (%) in *Plagiostachys austrosinensis* following experimental pollination treatments. GLM (Spss19) and MANOVA (Spss19) were used to compare fruit sets between cataflexistylous and anaflexistylous flowers; A value of *P* < 0.05 was accepted as significant difference; Similar superscript letters for each parameter indicate no significant difference at *P* < 0.05; *n* indicates the number of flowers; *N* indicates the number of stems

Morph	Open pollination (*n/N*)	Bagged flowers (*n/N*)	Experimentally outcrossed (*n/N*)	Experimentally selfed (*n/N*)	Anthers removed in bud (*n/N*)	Stigma removed in bud (*n/N*)
Cataflexistyly	22.56^a^ ± 10.69 (158/16)	–	21.43^a^ ± 9.34 (28/10)	21.33^a^ ± 7.95 (25/9)	– (25/5)	– (25/5)
Anaflexistyly	26.33^a^ ± 8.52 (125/11)	–	40.01^b^ ± 18.99 (34/9)	7.58^a^ ± 4.26 (25/11)	– (25/5)	– (25/5)

## Discussion

Takano et al. (2009) firstly reported the flexistyly in *Plagiostachys strobilifera*. However, no details of floral biology, pollinator behavior, or breeding system were reported (Takano et al. 2009). To our knowledge, this is the first study to demonstrate the pollination biology in a natural population of *P. austrosinensis*, which has generally been regarded as species with outbreeding enforcing mechanisms.

In this study, we found very low fruit set (7.58%) in experimentally selfed anaflexistylous flowers, demonstrating the presence of partial self‐incompatibility in this morph. Zhang et al. (2003) thought that self‐pollination in anaflexistylous plant did not happen under natural conditions as the stigma protruded beyond the anther after anther dehiscence. However, further studies of this and other species are needed, because inbreeding depression may be difficult to identify when it acts at a later stage of development or only under certain field conditions.

The P/O ratio is a better predictor of breeding system than other morphological characteristics (Cruden 1977). The P/O ratios of *P. austrosinensis* are 533.46 ± 92.37 (*n* = 10) in the cataflexistylous morph and 526.67 ± 101.36 (*n* = 10) in the anaflexistylous morph, suggesting an obligate xenogamous breeding system according to Cruden (1977). Automatic selfing (bagged flowers) treatments have no fruit set, showing that *P. austrosinensis* is an insect‐dependent species.

Selfed flowers have significantly lower fruit set than crossed flowers in the anaflexistylous morph, possibly due to inbreeding depression or partial self‐incompatibility. The male function of the anaflexistylous morph may be less efficient than that of the cataflexistylous morph, because the dispersal of self‐pollen from anaflexistylous flowers during the afternoon is prevented by the limited number of available female flowers on cataflexistylous plants. It means part of cataflexistylous plants may self‐pollinated in the morning already. On the other hand, the pollen robber (Mutillidae spp.) visiting frequency occurred from 1430 to 1530 h, staying on flowers for a significantly longer time than pollinators, which means more pollen were robbed as flower reward by pollen robber.

The period of nectar production is generally correlated with the period in which pollinators are active (Cruden et al. 1983). This is the case in *P. austrosinensis*. In our study, Vespidae spp. is considered to be an effective generalist pollinator; its peak visiting frequency occurs from 1430 to 1530 h, while the peak nectar volume of cataflexistylous and anaflexistylous flowers is reached at 1630 h. However, the daily variation in sugar concentration is relatively small.

Although the floral behavior is similar to that of the Chinese flexistylous *Alpinia* species reported previously, the pollinator visiting pattern is quite different. Takano et al. (2005) mentioned the differences of visiting pattern of pollinators between subtropics and tropics. In subtropics, the peak of flower visit is around noon. In tropics, the peak of flower visit is early morning and late afternoon. In this study, visiting frequency occurred from 1300 to 1600 h. It seems that the pollinator visiting pattern is adaptation to specific environmental conditions. On the other hand, we find certain kinds of pollens on pollinators' body (see Fig. 2E), showing that the pollinators are not species‐specific visitors. Much more ecological factors should be involved in the behavior of flower visitors.

In Takano et al. (2005) study, the flower visitors of *Alpinia nieuwenhuizii*, the carpenter bees, *Xylocopa latipes* and *Xylocopa collaris alboxantha*, are considered to be the most effective (and the only) pollinators. In our study, *Bombus pyrosoma* and Vespidae spp. are considered effective pollinators of *Plagiostachys austrosinensis*. Li et al. (2001a) found that the flower visitors of *Alpina kwangsiensis* were *xylocopid* spp. Zhang et al. (2003) found that the flower visitor of *Alpinia blepharocalyx* were *Apis cerana cerana* and *Xylocopa* spp.. It seems that the flower pollinators are also quite different. It would be worthwhile to determine the diversity of flower visitors associated with the evolution of flexistyly, which need to study in detail the reproductive biological traits in more flexistylous species.

Flexistyly is a floral dimorphism in angiosperms. It is the only sexual polymorphism that combines reciprocal herkogamy and heterodichogamy (Li et al. 2001a), which involves both spatial (herkogamy) and temporal (dichogamy) features of sexual function. The similar forms of reciprocal herkogamy and heterodichogamy occurs in at least dozens of unrelated families of flowering plants, which is generally considered to promote insect mediated cross‐pollination by reducing sexual interference between female and male function. Nevertheless, the basic reproductive biology of most sexual polymorphism species remains unstudied.

## Conflict of Interest

None declared.
